# Biotechnological Strategies for Cultured Poultry Meat Biofabrication Through Induced Pluripotent Stem Cell Reprogramming and CRISPR-Cas9-Mediated Genome Editing

**DOI:** 10.3390/ani16142193

**Published:** 2026-07-15

**Authors:** M Khuzema Niaz, Irtqa Hassan, Usama Abdullah, Malik Ahsan Ali, Nousheen Zahoor, Muhammad Mushahid, Hongyan Sun, Bichun Li, Kai Jin

**Affiliations:** 1 Joint International Research Laboratory of Agriculture and Agri-Product Safety, Ministry of Education of China, Yangzhou University, Yangzhou 225009, China; 2 Key Laboratory of Animal Breeding Reproduction and Molecular Design for Jiangsu Province, Yangzhou University, Yangzhou 225009, China; 3 College of Animal Science and Technology, Yangzhou University, Yangzhou 225009, China; 4 Constituent College Toba Tek Singh, University of Agriculture Faisalabad, Faisalabad 38000, Pakistan; 5 Institutes of Agricultural Science and Technology Development, Yangzhou University, Yangzhou 225009, China

**Keywords:** CRISPR-Cas9, induced pluripotent stem cells, cultured meat, cellular agriculture, *Gallus gallus*, avian genome editing, food security

## Abstract

The growing global demand for ethical, resource-efficient protein sources has renewed serious interest in cultured meat as a viable alternative to conventional livestock production. Two revolutionary biotechnological systems, induced pluripotent stem cell (iPSC) reprogramming and CRISPR-Cas9-mediated genome editing, when combined, offer unparalleled accuracy and scalability for the biofabrication of avian flesh. In this review, we present a comprehensive pipeline that involves the ectopic expression of Yamanaka factors (Oct4, Sox2, Klf4, and c-Myc) to reprogram primary somatic cells derived from *Gallus gallus* into induced pluripotent stem cells (iPSCs). This process is subsequently followed by targeted genome editing to enhance myogenic potential, growth efficiency, nutritional composition, and disease resistance. iPSCs are cultivated in a xeno-free bioreactor following genome editing, and subsequently directed to develop into myoblasts and mature myotubes. Three-dimensional tissue biofabrication is realized by combining biomaterial scaffolds and perfusion bioreactor systems, structuring an authentic muscle tissue matrix. These engineering platforms enable precise control over microenvironmental parameters, including oxygenation and nutrient perfusion. The resulting biofabricated poultry product is compositionally optimized, free of antibiotic residues, and exhibits a significantly reduced environmental footprint than poultry that is grown in the traditional way. This all-in-one solution solves important problems in food security, animal welfare, land use efficiency, and greenhouse gas emissions while also setting up a scalable biomanufacturing framework for making proteins for the next generation.

## Simple Summary

In this review, we present a conceptual pipeline that involves combining induced pluripotent stem cell reprogramming via Yamanaka factors with CRISPR-Cas9-mediated genome editing specifically for the biofabrication of cultured poultry meat, moving beyond isolated applications in mammalian or biomedical models. Unlike previous studies that focused separately on avian iPSC derivation or genome editing for disease resistance, this work delivers a complete, stepwise pipeline from somatic cell reprogramming in *Gallus gallus* to target the genetic enhancement of myogenic potential, growth efficiency, and nutritional composition, followed by xeno-free bioreactor expansion and 3D tissue biofabrication using perfusion systems. The proposed platform uniquely addresses scalability, antibiotic-free production, and carbon footprint reduction for poultry meat, providing a transformative biomanufacturing blueprint that bridges cellular agriculture, precision genome engineering, and sustainable food systems.

## 1. Introduction

The global food system faces a supply–demand mismatch that will only deepen over the coming decades. With the world population projected to approach 10 billion by 2050, per capita protein demand is rising fastest in precisely those regions where the infrastructure for conventional animal agriculture is already stretched [1]. Poultry is central to that equation. Chicken is, by a substantial margin, the most consumed meat globally, a position earned through a combination of efficient feed conversion, shorter production cycles relative to ruminants, lower land and water requirements per kilogram of output, and broad cross-cultural dietary acceptability [2]. As an affordable, high-quality source of complete protein and essential micronutrients, poultry plays a disproportionate role in food security across both rural and peri-urban populations worldwide [3].

Yet the systems delivering this poultry are under mounting structural pressure. Conventional selective breeding, for all its historical success, operates on generational timescales and is constrained by a narrowing pool of available genetic diversity in commercial lines [4]. Complex quantitative traits, heat tolerance, feed conversion efficiency, and resistance to rapidly evolving pathogens like highly pathogenic avian influenza are difficult to improve simultaneously through classical approaches, and the pace of environmental change is rendering some adaptations urgently necessary [5]. Beyond productivity, the environmental footprint of industrial poultry farming is well-documented: greenhouse gas emissions, land conversion, nitrogen loading, and water consumption are all significant, and the ethics of intensive confinement systems are attracting increasing public and regulatory scrutiny [6]. These are not peripheral concerns that incremental improvements can resolve; they are structural features of the conventional model. 

Two biotechnological developments, considered in combination, offer a substantively different approach. Induced pluripotent stem cell (iPSC) technology, described originally by Shinya Yamanaka and colleagues in 2006, established that differentiated adult somatic cells can be reprogrammed to a pluripotent state capable of generating virtually any cell type in the body [7]. The introduction describes several ways to make immortal cell lines for growing meat without using live animals. This gives researchers exact control over the ratio of muscle to fat cells [8]. In an agricultural context, this creates the possibility of establishing renewable, genetically stable cell lines from elite donor animals, circumventing the need for repeated slaughter in the production chain. Simultaneously, CRISPR-Cas9 genome editing has, since its characterization in 2012, reshaped what is practically achievable in precision genetic modification [9]. Unlike earlier transgenic approaches, which introduced foreign DNA at semi-random genomic locations with unpredictable positional effects, CRISPR enables targeted, defined edits from complete gene knockouts to single-nucleotide substitutions with a speed and cost profile that has made the technology accessible well beyond specialized genome engineering laboratories [10]. The strategic value of combining these two tools lies in their complementarity. iPSCs provide a stable, self-renewing cellular substrate on which CRISPR edits can be introduced, validated at the clonal level, and then directed toward specific functional lineages in the context of cultured meat, principally skeletal muscle and adipose tissue. This combination allows genetic modifications to be rigorously characterized in vitro before any consideration of germline transmission or large-scale production, providing both a scientific validation layer and a regulatory evidence base [11].

**Figure 1:**
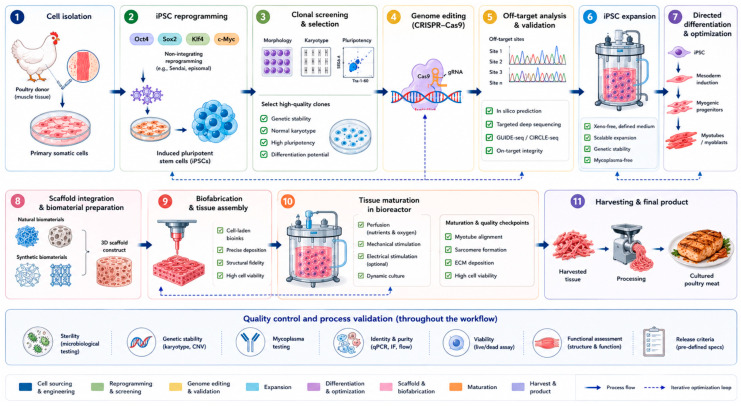
Schematic overview of the cultured poultry meat production workflow using induced pluripotent stem cell (iPSC) technology. The process includes poultry somatic cell isolation, iPSC reprogramming, clonal screening and selection, CRISPR-Cas9 genome editing with off-target validation, scalable iPSC expansion, directed myogenic differentiation, scaffold integration, biofabrication, bioreactor-mediated tissue maturation, and harvesting of cultured poultry meat. Quality control and process validation—including sterility, genetic stability, cell identity, viability, and functional assessment—are integrated throughout the manufacturing pipeline to ensure product safety, reproducibility, and scalability.

This review establishes an integrated biomanufacturing blueprint that directly addresses the unique biological and mechanical bottlenecks of avian systems. Moving beyond decoupled upstream engineering summaries, we analyze the specific metabolic crossroads, genetic stability vectors, and downstream texturing prerequisites required to translate avian cellular agriculture from a conceptual model into a scalable food matrix. Biosafety, animal welfare considerations, and the governance frameworks applicable to gene-edited food products are addressed throughout (Figure 1).

## 2. Review Methodology

To establish a comprehensive and rigorous scientific blueprint for the integration of avian stem cell engineering and precision genome editing, a structured literature search and evaluation process was performed. This approach ensures an objective synthesis of the decoupled upstream protocols and downstream texturing prerequisites detailed throughout this review. 

A structured literature search was conducted across four electronic databases—PubMed, Web of Science, Scopus, and Google Scholar—with the final search update completed in May 2026. Search terms were constructed in Boolean combinations using both controlled vocabulary and free-text keywords. The primary search strings included: “induced pluripotent stem cell*” or “iPSC” & “poultry”, “chicken”, “*Gallus gallus*”, “avian”; “CRISPR”, “CRISPR-Cas9” or “genome editing” & “cultured meat” or “cellular agriculture” or “meat quality” or “myogenesis” or “muscle growth”; “cultured meat or “cell-based meat” & “bioreactor” or “scaffold” or “3D bioprinting” or “tissue engineering” or “xeno-free”; & “primordial germ cell” & “reprogramming” or “pluripotency” or “germline competence”.

Articles published between January 2006, the year of the seminal iPSC publication, and May 2026 were considered for inclusion. Priority was given to peer-reviewed original research articles and systematic reviews in English. Book chapters, technical reports, and gray literature were included selectively where primary peer-reviewed coverage of a given topic, particularly regulatory and market-related content, was limited. Studies were excluded if they addressed mammalian cell lines only without relevance to the avian pipeline described here, reported purely in vitro toxicology without a biotechnology application angle, or lacked sufficient methodological transparency for critical evaluation. Reference lists of key review articles were hand-searched to capture relevant studies not returned by the database queries. Where conflicting findings between studies were identified particularly regarding avian iPSC pluripotency status and reprogramming efficiency, these are explicitly discussed rather than resolved by citation of the most convenient source.

## 3. Induced Pluripotent Stem Cells in Poultry

iPSCs represent one of the most important advances in modern regenerative biology and animal biotechnology [12]. Transcription factors are expressed in differentiated somatic cells to transform these cells into pluripotent cells. Once the iPS cells have been induced, they will be able to grow and differentiate into cells representing all three germ layers, as embryonic stem cells are capable [13]. In 2006, Shinya Yamanaka and his colleagues applied the technology of iPSCs. The method has since become expanded into biomedical and veterinary disciplines including remarkably poultry biotechnology [14]. The advent of the iPSC knowledge offers immense visions to innovation around genetics, preservation of germplasm, modeling of diseases, and generation of poultry meat in the context of poultry exploration. Although the study of iPSCs in birds is still in its beginnings and in comparison with mammals, we have achieved a lot of progress in the biology of pluripotent stem cells in birds and the techniques that they can be utilized in poultry farming [15].

### 3.1. Concept and Biological Characteristics of iPSCs

The iPSCs are genetically modified somatic cells that have been brought back to a pluripotent state to become many different cell types, also capable of producing more of themselves [16]. The Yamanaka factors are Oct4, Sox2, Klf4, and c-Myc. They stop differentiation that is specific to a certain lineage and encourage the activation of pathways that already exist for pluripotency. From a biological perspective, iPSCs and ESCs have numerous common traits [17]. These include a high proliferative rate, expression of pluripotency markers such as OCT4, SOX2, NANOG, and SSEA-1, and the ability to form embryoid bodies during in vitro differentiation [5]. Colonies that are tightly packed, have a high nucleus-to-cytoplasm ratio, and show significant alkaline phosphatase expression are indicative of iPSC morphology. These traits show that pluripotency has been reached [18].

Whereas mammals have pluripotent stem cells in their blastocysts, those in birds such as chickens and quail differ in some respects from mammals. For example, birds lack an inner cell mass stage in their development [19]. Pluripotent cells reside in the epiblastic layer of the developing avian embryo. The developmental heterogeneity observed during this era affects the maintenance of pluripotency and the stability of avian iPSCs cultivated in vitro. Despite the previously noted heterogeneity, considerable evidence demonstrates that avian iPSCs have a wide range of genetic and functional traits akin to mammalian pluripotent stem cells [20].

### 3.2. Methods for Generation and Maintenance of iPSCs

To make iPSCs in birds, transcription factors that promote pluripotency must be made to work by being put into somatic cells. Early studies indicate that using viral delivery systems, such as retroviral and lentiviral vectors, the OCT4, SOX2, KLF4, and c-MYC genes can be transferred to the fibroblasts of chickens and quails. This implies that the induction of pluripotency is shared in all organisms since the induced pluripotency stem cells (iPSCs) can be produced by using human pluripotency-inducing genes on quail fibroblasts [21]. The range of cell types utilized for reprogramming has been expanded due to successive inquiries into donor cell types. It is possible to make stem cell-like cells from fibroblasts in the skin, feather follicles, and developing fibroblasts from chickens [15]. The ease of access to feather papilla cells through non-aggressive testing makes them very useful for study that looks at significant breeding lines. 

To keep pluripotent stem cells pluripotent after production, they must be grown in a restricted environment. While bFGF and murine embryonic fibroblast (MEF) feeder layers are common for initial avian iPSC derivation, scaling for cultured meat production strictly requires the adaptation of these lines to fully xeno-free, suspension-capable media [22]. These situations delay natural variation while advancing endless cellular production. Furthermore, studies demonstrate that small molecule inhibitors of signal transduction trails, including TGF-β and Wnt/β-catenin, improve reprogramming ability and maintain pluripotency. Several tests are performed to confirm iPSC generation, including the assessment of pluripotency pointers, the development of embryoid constructions, and in vitro differentiation [23]. Molecular procedures including reverse transcription polymerase chain reaction (RT-PCR), immunofluorescence cataloging, and transcriptome analysis are commonly applied to confirm the expression of pluripotency-related genes in avian iPSCs [24].

### 3.3. Current Progress and Challenges in Avian (Poultry) iPSC Research

Over the last ten years, there has been a dramatic growth in the number of people who wish to study iPSCs that come from hen eggs. Researchers have conducted a lot of work on altering genomes using chicken iPSCs. Chickens are easy to work with, which is why they are applied as models for genetic engineering [25]. Changing the DNA of these cells before they differentiate can also make them better in ways that are desirable, including making them less likely to get sick or helping them grow quicker. 

Another significant function is to keep the bird groups’ genetic diversity safe. iPSCs can help chickens preserve their good genetic lines and bring back genetic diversity because they can be frozen [23]. Researchers have also explored using iPSCs from birds as a prospective supply of cells for produced meat. iPSCs can turn into any kind of cell, therefore, they could be employed in a lab to manufacture meat products by turning into muscle and fat lines. Some things have gotten better, but there are still many difficulties to fix. Reprogramming birds is not as effective as reprogramming mammals, and it might be challenging to sustain pluripotency for a long period [26]. It is more difficult to share information about stem cell techniques that have been proven to work in mammals since embryos in birds and mammals grow in different ways. Future studies ought to focus on improving the effectiveness of reprogramming, discovering pluripotency regulators specific to avian species, and establishing ideal conditions for the extended development of iPSCs [27]. New genomic technologies, such as CRISPR-based gene editing and single-cell transcriptomics, will probably speed up progress in this field and make it easier to apply iPSC technology in modern biotechnology and poultry breeding programs [28].

### 3.4. Comparative Analysis of Avian Cell Sources

Before detailing the iPSC pipeline, it is necessary to weigh this approach against other cell sources currently used in cultured meat research (Table 1). Primary somatic cells, such as satellite cells, remain the standard for early-stage proof-of-concept work. However, scaling up meat biofabrication requires cell lines that can multiply indefinitely and differentiate into multiple tissue types (muscle, fat, and connective tissue). Table 1 outlines how iPSCs compare with adult stem cells and other pluripotent lines regarding these specific industrial requirements.

## 4. CRISPR Gene Editing Technology

The CRISPR-associated protein (Cas) system is a new and exciting technique to modify the DNA of living things. It derives from how bacteria and archaea modify their immune systems to fight off viruses and plasmids. Guide RNAs (gRNAs), which are very tiny RNAs, tell the Cas nuclease protein (typically Cas9) where to go in the genome. It sticks to the DNA pattern that is the exact opposite of the one next to it. The Cas enzyme finds the sequence and then chops the DNA in half next to the PAM sequence, which stands for “protospacer neighboring motif”. Natural DNA repair mechanisms, including homology-dependent repair (HDR) and non-homologous end joining (NHEJ), correct double-strand breaks (DSBs). NHEJ is not very reliable; however, it does aid knockouts by adding or removing genes. Genetic sequences can be modified with a donor template if high-density random sampling (HDR) is employed. This enables the user to add or take away genes with great accuracy [29]. The CRISPR/Cas system quickly became the most popular approach to modify genomes in biology and farming biotechnology because it is easy to use, create, and works well. Researchers who work with cows and poultry have found that CRISPR technology makes it easier to change genes in several ways. Some of these include base editing, multiplex genome editing, knockout, and knock-in. These editing methods can change genes that influence how well something works, how resistant it is to disease, and how good it is [16]. Gene knockout advances are typically used to stop genes that give people poor qualities or make them more likely to get sick. Gene knock-in advances, on the other hand, use HDR-mediated repair processes to add good alleles or genes that work to select sections of the genome. Base editing and prime editing are two new, more powerful approaches that use CRISPR as a starting point. With these technologies, accurate nucleotide replacements can be made without breaking the double strand. This stops modifications from happening that do not need to materialize. Another thing about CRISPR is that it can change multiple things at once, which means that it can modify many genes at once by using a lot of guide RNAs. This is highly effective for modifying features in animals that are regulated by more than one gene [30].

**Figure 2:**
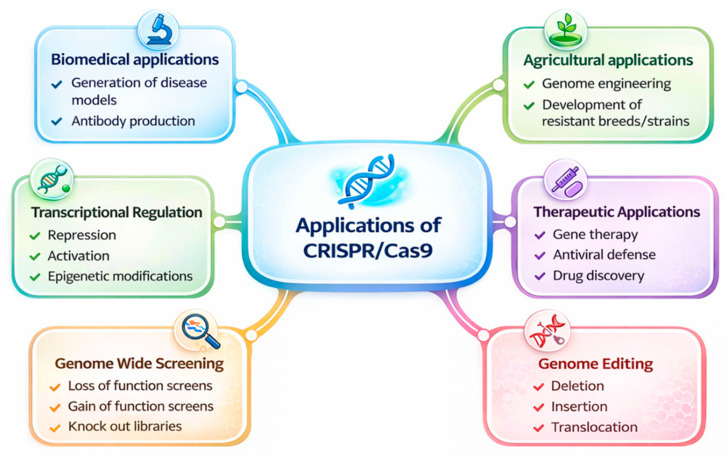
Overview of the major applications of CRISPR/Cas9 technology across different fields. The system enables precise genome editing through gene deletion, insertion, and translocation, and supports genome-wide functional screening such as loss- and gain-of-function studies. CRISPR/Cas9 is widely used in transcriptional regulation, biomedical research for disease modeling and antibody production, therapeutic development including gene therapy and antiviral strategies, agricultural biotechnology for genome engineering, and the development of improved or disease-resistant animal breeds.

CRISPR is a better technique to change genes than zinc-finger nucleases (ZFNs) and transcription activator-like effector nucleases (TALENs). A few of these advantages include increased editing options, reduced expenses, simplified design, and additional methods for targeting genomic loci [8]. It is possible that introducing random foreign DNA to the genome is a normal element of transgenic procedures. This could lead to changes in gene expression or unexpected genetic effects. CRISPR is a better and more valuable technology for genetic planning projects since it can edit specific sections of the genome [31]. Instead of having to breed for many cohorts, it is also possible to make alterations to genes in just one generation, which speeds up the process of designing better animal lines. This is why gene editing using CRISPR has transformed how people rear poultry and cows these days. This could help the world’s food security challenges by making more meat, making animals healthier, and more [32] (Figure 2).

### CRISPR Variants and Their Relevance to the Avian Cultured Meat Pipeline

A significant evolution has occurred within the CRISPR toolkit since the initial characterization of Cas9-mediated genome editing. Selecting the appropriate editing modality for a given target is not a trivial choice, as it directly affects the editing precision, the nature and risk of off-target effects, the need for homology-directed repair templates, and ultimately, the regulatory evidence burden associated with the edited cell line. For a cultured meat pipeline, where genomic safety for human consumption is a central concern, this selection matters considerably. Table 2 summarizes the principal CRISPR variants and their relative merits for the specific applications proposed in this review (Table 2).

## 5. Integration of iPSCs and CRISPR for Poultry Meat Production

Combining iPSCs with CRISPR genome editing technology could make it faster to change the genetics of chicken meat production. Because they can make new copies of themselves and change into different types of cells from the three germ layers, such as skeletal muscle cells, iPSCs are ideal biological platforms for genetic engineering and developmental investigations in cattle [33]. iPSCs make a controlled zone where changes to individual genes can be made before passing them on to future generations or changing them. When used with CRISPR-based genome editing, this works best. Rather than rehashing broad baseline cell bottlenecks, this setup targets the poorly understood molecular dynamics governing avian-specific blastoderm epiblast pluripotency networks within long-term fluid bioreactors [34]. Applying these combined technologies directly supports functional genomics and targeted cell line improvement. For cultured meat, this means that we can selectively edit the genes that control in vitro muscle growth, metabolic efficiency, and culture stability [35]. For example, knocking out the myostatin (MSTN) gene removes the natural brakes on skeletal muscle hyperplasia, ultimately maximizing muscle yield inside a bioreactor. Similarly, upregulating key myogenic drivers like MYOD, MYF5, and PAX7 can help streamline complex differentiation protocols [36]. We can also edit pathways linked to cellular metabolism, making the cells hardier against the physiological stresses of large-scale perfusion culture. While CRISPR editing of primordial germ cells (PGCs) remains highly relevant for breeding disease-resistant live poultry flocks, its primary value for cellular agriculture lies in generating robust, self-renewing cell lines purpose-built for bioreactor environments [34].

After genome editing, the controlled differentiation of converted iPSCs into skeletal muscle cells is a crucial step in understanding how genes work and making more fowl meat. It is possible to drive iPSCs to develop into muscle progenitor cells by specifically activating signaling pathways that replicate embryonic myogenesis. During this phase, myoblasts expand under the regulatory control of PAX7 and downstream myogenic factors. While mammalian protocols are highly robust, direct, high-efficiency conversion of chicken iPSCs into mature multinucleated myotubes remains historically limited by lineage-commitment gaps; thus, optimizing species-specific avian transcription cues remains a critical research frontier. Directed differentiation approaches often involve signaling molecules and growth factors to assist the mesoderm development and myogenic lineages become more specialized. Scientists can use these systems to learn about how particular genetic modifications affect how muscle cells are formed, how they grow, and how they use energy. Modified iPSCs can be used to manufacture muscle cells that could help scientists learn more about how muscles work and improve technology for producing chickens that makes it easier to grow muscle tissue in a lab. This would lead to more sustainable meat production [37,38,39]. 

The study has found a novel technique to use biotechnology in chickens that combines CRISPR gene editing with induced pluripotent stem cell (iPSC) technology. This approach accelerates the finding and establishment of beneficial genetic traits with greater precision. Such traits, including enhanced muscle yield, superior myofiber organization, improved feed conversion, and resistance to specific pathogens, are precisely the targets where the iPSC–CRISPR platform offers its greatest practical advantage over conventional breeding, given the precision and speed with which individual genetic modifications can be introduced, validated, and characterized in a defined cellular environment [7]. Ongoing research is advancing these technologies, suggesting that CRISPR-edited iPSCs may be essential for the future development of high-yield and sustainable poultry production systems, despite current technical challenges including stable germline transmission and efficient avian stem cell derivation [40,41].

## 6. Applications of iPSCs and CRISPR in Poultry Meat Production

Recent advancements in genome engineering and stem cell research offer likely prospects for refining the meat production abilities of chicken. Notable innovations encompass the application of CRISPR gene-editing technology and iPSCs, which facilitate genetic alteration for prospective benefits [42]. These technologies allow accurate modifications to be made to the genes that can control growth, muscle development, feed efficiency, and disease resistance, all of which are significant factors in determining the meat yield and quality of poultry production systems [24].

Among the applications of CRISPR technology that can have the most significant impact on the meat production process, the improvement of growth rates and muscle-building in chickens should be mentioned. Researchers can possibly boost the carcass yield and meat quality by making a few small adjustments to genes that regulate muscle growth processes. Primordial germ cell (PGC)-mediated genome editing has been successful in chickens with CRISPR-based genome editing, which has allowed for the generation of genetically modified chickens with inherited characteristics. With the help of this technique, it is possible to modify genes associated with growth control, metabolism, and skeletal muscle formation precisely. Such genetic modifications can enhance the overall productivity and feed conversion efficiency of broiler chickens [43]. However, achieving a consumer-accepted final product necessitates expanding beyond pure myocyte biomass. Downstream processing must incorporate adipocyte co-cultures to replicate natural poultry lipid distributions, introduce metabolic pathways favoring volatile flavor precursors for cooking aroma, and optimize cross-linking scaffolding mechanics to simulate authentic chicken myofibrillar textures. Also, CRISPR has been applied to create more resistant chicken lines to infectious diseases, such as avian influenza, which has a considerable impact on the poultry industry worldwide. Experiments showed that CRISPR-edited hens were more resistant to viral infection, which suggests that genome editing can be used to improve flock productivity and health [44]. 

In addition, iPSCs offer an advantageous basis to the development of poultry biotechnology and meat production. Somatic cells that have been subjected to pluripotent reprogramming are called iPSCs. These cells have the potential to differentiate into various types of cells, such as muscle and adipose tissue cells. Avian developmental biology, gene-function, and cellular differentiation machinery studies of muscle formation could be improved with the help of iPSCs. Genetic modification of these cells with CRISPR can also be followed by the production of transgenic chicken lines that have better production characteristics. Recent reports that have managed to produce chicken iPSCs using somatic cells in combinations of transcription factors such as OCT4, SOX2, NANOG and KLF4 have demonstrated that it is possible to produce persistent pluripotent cell lines in avian species [40,45]. 

The other interesting application of iPSC technology to poultry farming is the possibility of cell-based or cultured meat production. To transition these cells into food, engineering systems must scale up using specialized microcarrier selection inside stirred-tank bioreactor setups. This requires hydrogel-based scaffolding arrays optimized for anchorage-dependent cell attachment, alongside precise perfusion parameters to protect fragile avian cell lines from mechanical shear-stress damage during continuous culture operations. These methods may be used to meet the increasing global need for animal protein and lessen the adverse environmental impacts of traditional livestock farming. Moreover, the systems of iPSCs can be used to offer valuable experimental models that can be used to investigate muscle growth, metabolic signaling, and the genetic regulation of poultry meat quality traits [40]. Looking forward, coupling CRISPR with iPSC technology offers a highly precise toolset for cellular agriculture. Once iPSCs are modified, the resulting clonal lines can be validated for specific biomanufacturing traits such as faster in vitro growth, better nutrient uptake in synthetic media, and reliable myogenic differentiation [25]. This setup allows researchers to test gene functions in a controlled cell environment before committing to expensive scale-up phases. While this technology theoretically holds the potential to accelerate traditional poultry breeding by bypassing generational crosses, its most immediate and practical application is the optimization of isolated cell lines for industrial meat production [24]. 

Despite these promising applications, several challenges remain to be solved before these technologies can be widely implemented in commercial poultry production [46]. The enhancement of the efficiency of avian stem cell reprogramming, the stability of the transmission of altered genes to the germline, reduction in the off-target effects of genome editing, and the resolution of ethical and legal issues related to genetically modified animals are some of these challenges. However, ongoing research is progressing CRISPR and iPSC technologies, which indicates that these technologies will gain relevance in the next few decades in terms of sustainable chicken production and global food security [32,40].

## 7. Challenges and Limitations

Although CRISPR and iPSC technologies have huge potential in the production of poultry meat, their practical usage remains limited due to the entrenched technological issues. The fact that the chicken zygote is embedded in a large mass of yolk containing a small germinal disc also poses a fundamental challenge to the avian reproductive system, such that direct embryo microinjection, the classical approach to mammalian genome editing, is mostly impractical [34]. As a result, CRISPR/Cas9 gene-editing methods for birds rely on the isolation, in vitro growth, and proliferation of primordial germ cells (PGCs), which are subsequently genetically altered and injected into the bloodstream of growing recipient embryos. The efficiency of creating genome-edited chickens is greatly decreased by this multi-step process [47]. Mutational rates, such as on-target insertion and deletions, large deletions, and off-target mutations, can differ substantially between cell types of chickens, delivery vectors, and genome-editing techniques because of variation in cellular metabolism, efficiency of the DNA repair mechanisms, chromatin openness, and time of Cas9 expression. The process of obtaining iPSCs from avians is much more complicated. Though lots of various OSKM gene cocktails employing viruses, transposons, or minicircles successfully produced totally pluripotent cells, germline competence in avian iPSCs has not been shown yet. Nevertheless, the study of iPSCs in avians demands additional investigation than their study in humans and rodents [16,39]. In addition, the stability and efficacy of germline genome editing remain critical issues when viral vectors and cell transfer technologies are utilized in other types of cells, including ESCs, SSCs, and iPSCs; further species-specific studies must be conducted to optimize the outcomes. Moreover, outside of experimental conditions, economic and safety factors represent major barriers to large-scale poultry meat manufacturing, starting from the proof-of-concept to industrial-level production. Type-dependent genome editing and off-target effects, particularly in the case of stem-like cells, present substantial difficulties in developing an industrial application of cell line generation using CRISPR technology [48]. Beyond cellular biology, the lack of standardized global regulations for cultured meat creates significant market confusion. Furthermore, environmental sustainability claims remain highly uncertain in current life cycle assessments (LCAs). While land and water footprints are undeniably reduced, large-scale cell facilities demand massive electricity inputs to maintain bioreactor temperatures and run ultra-purification systems for media components. These energy demands may offset greenhouse gas savings unless the facilities are powered by fully renewable grids, presenting a major challenge for the global market [47]. When considered collectively, these deeply interconnected technical, safety, and economic challenges highlight that iPSC reprogramming and CRISPR editing in poultry will remain out of reach for commercial meat production without significant advances in delivery efficiency, cell line stability, low-cost basal media formulation, and regulatory harmonization across jurisdictions [49].

### The Regulatory Landscape: Where the Pipeline Actually Has to Land

Technical progress in the laboratory only translates to commercial impact if it can navigate the regulatory frameworks governing genetically modified organisms and novel food products, and these frameworks vary substantially by jurisdiction, are still being actively developed in most countries, and will look considerably more demanding when applied to CRISPR-edited iPSC-derived products than to the primary myoblast-based products that have so far received approvals.

The United States operates a dual-oversight system in which the FDA evaluates the safety of the cell lines and production process, while the USDA oversees labelling and market-entry requirements. This model has now been proven functional: as of early 2026, five cultured meat products have received full regulatory clearance in the U.S., including chicken from Upside Foods and GOOD Meat demonstrating an established procedural pathway rather than a hypothetical one. However, the involvement of CRISPR-edited iPSCs, as opposed to unmodified primary cells, would trigger additional scrutiny at the FDA level, requiring a demonstrable safety dossier for each genomic modification introduced including WGS evidence that off-target editing events are absent from the approved cell line used in production. The regulatory data package would essentially need to include the complete genomic characterization of the specific iPSC clone from which the production line is derived, not just the intended modification.

In the European Union, cultured meat falls under the Novel Food Regulation (EU) 2015/2283, with safety evaluation conducted by the European Food Safety Authority (EFSA). The EU pathway is materially more conservative than the U.S. approach, and as of early 2026, no cultured meat product has received EU market authorization. The additional regulatory layer created by the EU’s evolving framework for New Genomic Techniques (NGTs) introduces further complexity. Unedited cell biomass follows a standard Novel Foods pathway, whereas CRISPR-edited lines trigger distinct legal evaluations. For instance, single gene knockouts (e.g., MSTN) may be classified under a lighter compliance burden, whereas complex gene insertions require deep, multi-generational genotoxicity validation. The current legislative proposal distinguishes NGT Category 1 product modifications equivalent in scope to those achievable through natural variation or conventional breeding from NGT Category 2 products, which carry a heavier evidence burden. Whether CRISPR-edited iPSC lines for cultured poultry production would be classified under Category 1 or 2 remains, at the time of writing, an open regulatory question, and one with significant commercial implications.

Singapore remains the clearest market for the first commercial entry with approved products already in distribution, having authorized cultured meat from three producers by late 2025. China has invested substantially in cellular agriculture infrastructure and is developing its own regulatory framework, but formal guidance specific to CRISPR-edited cell lines for food use had not yet been issued at the time of this review. Across all jurisdictions, one requirement is consistent: comprehensive genomic safety data, including the whole-genome sequencing of edited cell lines, is a non-negotiable component of any regulatory submission. This must be treated not as a final validation step, but as an integral part of the experimental pipeline described throughout this review.

## 8. Future Perspectives

Modern biotechnology will no doubt be a driving force in shaping both the future availability and sustainability of poultry meat production, particularly as CRISPR-Cas9 gene editing and iPSC technology find increasing application. There have been significant advances in the field, but considerable gaps and challenges remain amongst these, including the increased efficiency and reliability of avian stem cell reprogramming, sustainable targeting of genome-edited chickens to germlines combined with stable integrations, and the accumulation of evidence required to meet the regulatory demands as well as societal acceptance for genetically modified (GM) and derived food products overall [24]. A growing body of research is increasingly turning its attention toward precision genome editing, the creation of poultry lines with built-in disease resistance and improved growth performance, and the harnessing of genomic data alongside advanced bioinformatics tools, all in the pursuit of more productive and sustainable poultry systems [50]. Meanwhile, the poultry sector around the world is paying more attention to these modernizations. This is attributable to their capacity to advance animal health, upgrade feed efficiency, and adjust the overall production performance, as both stem cell-derived meat and gene-edited chicken are likely to generate similar regulatory consequences [51]. However, successful commercialization will depend on regulatory approval, consumer acceptance, and cost-effective production strategies [35]. In the future, modern biotechnology is predicted to surpass traditional breeding methods, facilitating precise genetic alterations, enhancing sustainable poultry production systems, and fostering the creation of alternative protein sources, such as cultured chicken meat. These technologies could significantly meet the global demand for high-quality animal protein while simultaneously reducing the environmental impacts associated with conventional production methods.

## 9. Conclusions

In conclusion, advances in CRISPR-Cas9 gene editing and iPSC technologies have made it possible for chicken meat to be produced. These new technologies make precise genome editing feasible, and the application of the latest cellular technologies possible. The integration of the two approaches provides a robust and versatile platform to produce genetically enhanced avian cell lines that can develop into skeletal muscle cells. Translating this from a laboratory model to a scalable cellular agriculture pipeline requires overcoming severe technical and economic bottlenecks on the efficiency of the poultry industry, the development of biomedical research, and the growth of poultry meat produced from cells as an important component of the burgeoning cellular agriculture industry. Despite all of the advances, there are still many challenges to be addressed in terms of the efficiency of the editing, regulatory approval, ethical concerns, and consumer acceptance of the gene-edited and cell-based chicken products. To bring these research breakthroughs to the market, we need more studies, advanced technologies, and transparent regulations. This partnership may be the key to revolutionizing chicken farming to make it more efficient, sustainable, and humane at a time when we need high-quality protein like never before. Success in this field relies on transparent regulatory harmonization and solving the fundamental bioengineering challenges of mass tissue production rather than theoretical genetic potential. 

## Figures and Tables

**Table 1:** Comparative analysis of cell sources for cultured poultry meat.

Cell Type	Proliferation Potential	Differentiation Capacity	Primary Advantages for Cultured Meat	Key Technical Limitations
**Satellite Cells (Primary Myoblasts)**	Limited (senescence occurs without immortalization)	Unilineage (highly efficient myogenic commitment)	High differentiation efficiency; well-established protocols.	Requires continuous scaffolding; incapable of producing adipocytes (fat).
**Mesenchymal Stem Cells (MSCs)/FAPs**	Moderate to High	Multilineage (adipogenic, fibrogenic, osteogenic)	Excellent source for generating the fat/lipid profile of meat.	Requires complex co-culture with myogenic cells to form whole-cut meat.
**Avian iPSCs**	Indefinite (inherent self-renewal)	Pluripotent (muscle, fat, connective)	Single-cell source for all meat components; highly scalable.	Complex, multi-step differentiation protocols; high regulatory burden.
**Embryonic Stem Cells (ESCs)**	High	Pluripotent	Genetically stable pluripotency.	Severe technical difficulties in isolating and maintaining true avian ESCs.
**Primordial Germ Cells (PGCs)**	Moderate to High	Germline-restricted	Established protocols for transgenic poultry breeding.	Not optimized or suitable for bulk somatic (muscle/fat) tissue engineering.

**Table 2:** Comparative overview of CRISPR editing variants for cultured poultry meat applications.

CRISPR Variant	Mechanism	DSB Required	Off-Target Risk	Best-Suited Application in Pipeline	Regulatory Status in Food Context
SpCas9 (wild-type)	RNA-guided double-strand break	Yes	Moderate–High	*MSTN* knockout via NHEJ; bulk gene disruption	Established; most regulatory precedent
eSpCas9/HiFi Cas9	Engineered high-specificity variants	Yes	Low	Precision knock-in at productive loci	Increasingly accepted; preferred over WT for food applications
Cas12a (Cpf1)	Staggered DSB; T-rich PAM preference	Yes	Low	Editing in AT-rich avian genomic regions	Limited poultry-specific data; growing interest
Adenine Base Editor (ABE)	A·T → G·C conversion; no DSB	No (nick only)	Very Low	Disease resistance allele introduction; subtle regulatory edits	Favorable profile; no DSB reduces genotoxicity concern
Cytosine Base Editor (CBE)	C·G → T·A conversion; no DSB	No (nick only)	Low–Moderate	Fatty acid pathway modification for nutritional enhancement	Favorable; used in agricultural species; avian data emerging
Prime Editor (PE)	Reverse transcriptase-guided; all 12 substitution types	No (nick only)	Very Low	Precise insertions and complex edits where no natural variant exist	Emerging; avian-specific validation studies still limited

## Data Availability

No new data were created or analyzed in this study.

## Abbreviations

iPSC	Induced Pluripotent Stem Cell
CRISPR	Clustered Regularly Interspaced Short Palindromic Repeats
Cas	CRISPR-associated protein
PGCs	Primordial Germ Cells
GM	Genetically Modified
TALENs	Transcription Activator-Like Effector Nucleases
PE	Prime Editor
ESCs	Embryonic Stem Cells
TGF-β	Transforming Growth Factor Beta
Wnt/β-catenin	Wingless/Integrated Beta-Catenin Pathway
RT-PCR	Reverse Transcription Polymerase Chain Reaction
MSTN	Myostatin
